# Highly Portable, Sensor-Based System for Human Fall Monitoring

**DOI:** 10.3390/s17092096

**Published:** 2017-09-13

**Authors:** Aihua Mao, Xuedong Ma, Yinan He, Jie Luo

**Affiliations:** 1 School of Computer Science & Engineering, South China University of Technology, Guangzhou 510006, China; heyinanscut@outlook.com; 2 School of Electronic and Information Engineering, South China University of Technology, Guangzhou 510006, China; eemaxuedong@mail.scut.edu.cn; 3 School of Fine Art and Artistic Design, Guangzhou University, Guangzhou 510006, China

**Keywords:** fall detection, sensors, easy portability, body segment

## Abstract

Falls are a very dangerous situation especially among elderly people, because they may lead to fractures, concussion, and other injuries. Without timely rescue, falls may even endanger their lives. The existing optical sensor-based fall monitoring systems have some disadvantages, such as limited monitoring range and inconvenience to carry for users. Furthermore, the fall detection system based only on an accelerometer often mistakenly determines some activities of daily living (ADL) as falls, leading to low accuracy in fall detection. We propose a human fall monitoring system consisting of a highly portable sensor unit including a triaxis accelerometer, a triaxis gyroscope, and a triaxis magnetometer, and a mobile phone. With the data from these sensors, we obtain the acceleration and Euler angle (yaw, pitch, and roll), which represents the orientation of the user’s body. Then, a proposed fall detection algorithm was used to detect falls based on the acceleration and Euler angle. With this monitoring system, we design a series of simulated falls and ADL and conduct the experiment by placing the sensors on the shoulder, waist, and foot of the subjects. Through the experiment, we re-identify the threshold of acceleration for accurate fall detection and verify the best body location to place the sensors by comparing the detection performance on different body segments. We also compared this monitoring system with other similar works and found that better fall detection accuracy and portability can be achieved by our system.

## 1. Introduction

The aging society is becoming a serious public health issue in many areas, especially in developed countries. According to the World Health Organization, falls are the second leading cause of accidental or unintentional injury deaths worldwide [1], and more than one third of elderly people fall once or more each year [2]. For this group of people, once they fall, they may suffer serious health problems. Damage may be greatly reduced if they have access to timely rescue. Thus, a reliable fall monitoring system has great application value and development prospects.

In the last decade, many methods have been developed for fall detection; most of which are based on optical sensors [3,4,5,6], accelerometers [7,8,9,10,11], and accelerometers combined with gyroscopes [12,13,14]. The principle of the optical sensor-based method is to capture the image by visual sensors, such as digital cameras and Kinect, and then distinguishes the human body with other items using digital image processing algorithms to detect falls. For example, Yang et al. [15] used a Kinect sensor to capture three-dimensional (3D) depth images and the dense spatio-temporal context (STC). The dense STC algorithm was applied to track the head position. They calculated the distance between the head and floor plane. If the distance is lower than an adaptive threshold, then the centroid height of the human was used to determine whether a fall accident occurred. Gasparrini et al. [16] proposed a fall detection method for indoor environments based on a Kinect sensor. In this method, an Ad hoc segmentation algorithm was used to analyze the raw depth data from the Kinect and recognize all the elements captured in the depth scene. The system extracts the elements and classifies blobs in the scene to recognize human subjects among the blobs, followed by a tracking algorithm between different frames. Lee et al. [17] used shape feature variation extracted from the captured image to detect abnormal events, such as fall, and utilized a shadow removal algorithm to improve the accuracy of object detection and tracking. Anderson et al. [18] recognized the silhouette of individuals from the image captured by cameras and then extracted the features and trained hidden Markov models to recognize future performances of these known activities, such as fall. Rougher et al. [19] used a single camera to obtain images and obtained the 3D trajectory of the human head and then distinguished fall from activities of daily living (ADL) by the trajectory. Methods based on optical sensors have high reliability and accuracy. However, their monitoring range is limited. Once the users leave the captured range of the picture or are blocked by other objects, falls cannot be detected. Furthermore, intensive computation is required to finish the detection, leading to difficulty in real-time monitoring.

Accelerometer-based methods measure acceleration of the human body by an accelerometer, and falls are detected based on a threshold value of acceleration. For example, Mario et al. [20] proposed an algorithm to detect human basic movements, such as fall events from wearable measured acceleration data. The algorithm was designed to minimize computational requirements while achieving acceptable accuracy levels based on characterizing some particular points in the temporal series obtained from a single sensor. Hsieh et al. [21] reported a novel hierarchical fall detection algorithm based on the measured acceleration data involving threshold-based and knowledge-based approaches to detect a fall event. Kurniawan et al. [22] used a triaxis accelerometer to obtain the acceleration of the human body and then determined whether the fall occurs depending on the acceleration. Albert et al. [23] used the accelerometer of the mobile phone to obtain the acceleration data, which is applied in the machine learning classifier to detect falls and classify the type of falls. These methods are not limited by the monitoring space and are not vulnerable to external interference. However, they used only acceleration data, which often mistakenly determine ADL, such as jumping and running, as a fall event and then trigger a false fall alarm. Thus, the accuracy of this kind of method may be affected if it is only based on an accelerometer. Thus, to improve the accuracy, an alternative approach is to combine a gyroscope and even a magnetometer with the accelerometer to work together. Pierleoni et al. [24] fixed an inertial unit on the waist of a human body by a belt to obtain the acceleration and the attitude angle and then detect the falls. However, they can only send the detection result to a nearby device via Bluetooth. If nobody is nearby, the fall alarm would not be noticed by other people. Wibisono et al. [25] used an accelerometer and a gyroscope embedded in a mobile phone to obtain the acceleration and angular velocity of the human body. A threshold-based algorithm was applied to detect falls based on the acceleration and angular velocity. However, this kind of method based on the mobile phone required the mobile phone provided with acceleration and gyroscope, which is not available in some mobile phones. Besides that, the weight of most mobile phones is far greater than that of the sensor devices and may lead to discomfort when carried for prolonged periods. Furthermore, though the mobile phone now is a necessity in daily life, it is not practical to fix it on a body location all the time. He et al. [26] introduced a fall detection and alerting system consisting of a smart phone and a customized vest integrated with a triaxial accelerometer and a gyroscope. The raw triaxial gyroscope and accelerometer data are preprocessed by a program running on the smart phone by the Kalman filter to reduce the noise measurement from the sensors. Then, a Bayes network classifier was used to determine whether the individual falls or not. The system only considers four typical subcategories of ADLs and two kinds of falls and only tried the sensors on the vest near to the neck. Ntalampiras et al. [27] proposed a learning mechanism based on hidden Markov models for automatic recognition of physical activities of humans and assessed the performance based on previous datasets from an accelerometer and a gyroscope. However, the performance of this kind of method based on machine learning is dependent on the training dataset.

In this study, we propose a highly portable, sensor-based system for monitoring human falls. A small self-packaged sensor unit and a mobile phone composed this system. The sensor unit includes a triaxis accelerometer, a triaxis gyroscope, and a triaxis magnetometer, which can be easily ported by the user to obtain the root mean square (RMS) of acceleration ($RMS=\sqrt{\left(Ax^{2}+Ay^{2}+Az^{2}\right)}$, *A_x_*, *A_y_*, and *A_z_* are the *x*-axis, *y*-axis, and *z*-axis acceleration) and orientation of the user. All the sensor data are sent to the mobile phone via Bluetooth communication and is further processed by a fall detection algorithm. If falls are detected and recognized, the mobile phone will issue an alarm and automatically call the emergency contact for timely rescue. To determine the threshold of acceleration for accurate fall detection and verify the best body location to place the sensors, we designed seven fall activities and nine ADLs and conduct the fall detection experiment by placing the sensor unit on the subjects’ shoulder, waist, and foot, respectively. We also compare this system with other similar works in terms of accuracy, portability, and comfort. Our system has good accuracy and is highly portable due to the components of a small sensor unit and a mobile phone. Furthermore, our system is comfortable to the user even if used for a long time.

The main contributions of this study include the following:

1. We design a highly portable fall monitoring system, which only consists a small self-packaged sensor unit and a mobile phone to detect falls, and a fall alarm can be automatically issued to the emergency contact to seek timely rescue. Furthermore, this system has great detection accuracy for both falling and ADL.
2. We re-identify an acceleration threshold of 2.3 g by experimental observation for accurate fall detection, which is effective to avoid determining ADL as falls, and thus improving the accuracy of fall detection. Benefitting from this new threshold, our system can achieve better accuracy compared with other similar works.
3. We verify the best location of the human body to wear the sensor by conducting a series of experiments and comparison of the performance of fall detection when the sensor unit is placed on different body segments. The waist is the best one for sensor placement where the detection accuracy is 100% without any misjudgment, whereas wearing the sensor in the shoulder and foot may still mistakenly identify ADL as falls.

## 2. Fall Monitoring System

**Figure 1:**
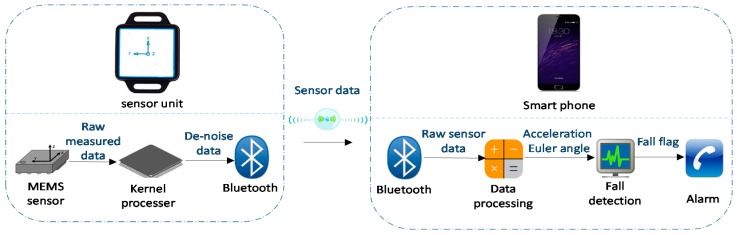
Highly portable, sensor-based fall monitoring system. MEMS: microelectrometrical system.

The system consists of a small self-packaged sensor unit and a mobile phone with an Android operating system (Figure 1). The sensor unit is easily carried by the user, containing a microelectrometrical system (MEMS) sensor, a kernel processer, and a Bluetooth module. The sensors’ data are transmitted to the Android mobile phone via Bluetooth. A developed application (APP) runs on the Android system to receive sensor data from the sensor device and processes the raw data to calculate the orientation and acceleration. Based on the orientation and RMS, the APP automatically detects falls based on a proposed fall detection algorithm. Once the fall is detected, an alarm is issued by the mobile phone and a call will automatically connect to the emergency contact.

**Figure 2:**
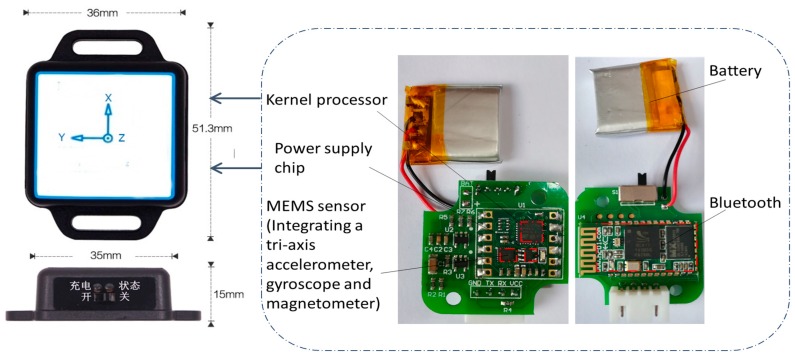
Small, self-packaged sensor unit.

Figure 2 shows the internal details of the self-packaged sensor unit, wherein the MEMS sensor module integrates a triaxis accelerometer at the range of ±16 g, a triaxis gyroscope at the range of ±2000°, and a triaxis magnetometer. The Kernel processor can reduce the measurement noise from the sensor by a filtering algorithm setup in the processor. The Bluetooth module includes a BC417 Bluetooth chip based on Bluetooth 2.0, and the range of the Bluetooth module is 15 m. The sampling frequency of the sensors from the human activities is set to 100 Hz. After noise reduction, the sampling data from the sensors are transmitted to the mobile phone via Bluetooth communication. The power supply module contains a power supply chip and a battery. With the power supply module, the device can work without any additional power after charging the battery. Thus, the device is very convenient to be carried by the users.

**Figure 3:**
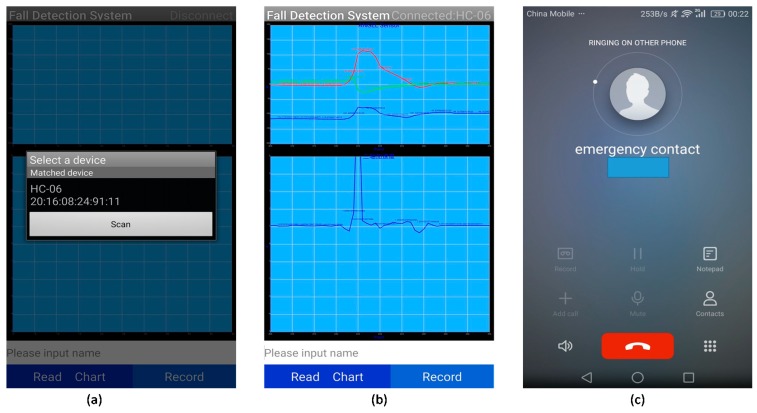
Main interfaces of the Android application: (**a**) device search; (**b**) data visualization and fall monitor; (**c**) making alarm and automatic calling.

Figure 3 shows the main interfaces of the Android APP. First, the APP searches for nearby Bluetooth devices and connects to the sensor unit. After establishing the connection and receiving the sensor data, the APP calculates the Euler angle and then visualizes the Euler angle and acceleration by data charts. Meanwhile, a fall detection algorithm was used to detect falls in real time. If a fall is detected, the APP will make an alarm sound and issue a call to the emergency contact.

## 3. 3D Orientation Measurement of the Human Body

**Figure 4:**
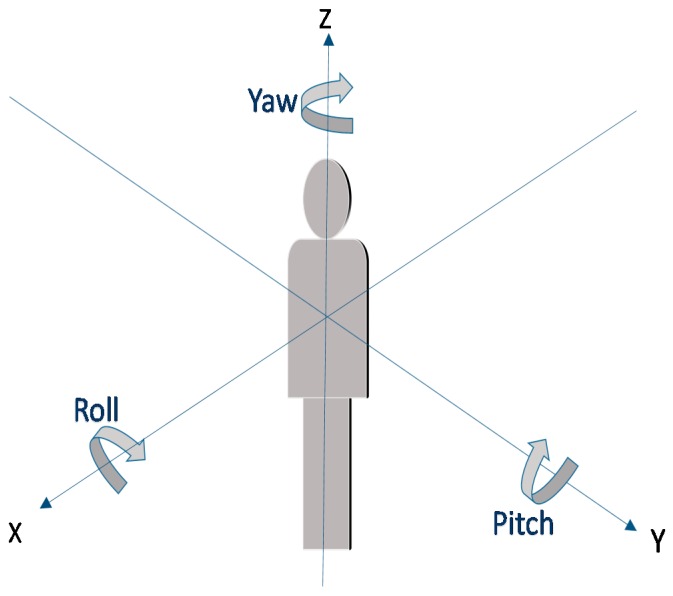
Pitch, roll, and yaw angle of the human body.

To describe the posture of the human body, we use the Euler angle, which is described by yaw, pitch, and roll angles to represent the body’s spatial orientation as shown in Figure 4. In the ideal case, a single 3-axis gyroscope placed on a rigid body can provide the current orientation of the body by performing integration calculation on gyroscope data. However, in practice, integration will accumulate the white noise from the reading effects, resulting in drift from the expected values. To complement the drift gyroscope data, sophisticated algorithms often fuse the accelerometer and magnetometer data due to their compensable characteristics. The performance of sensor data fusion is often determined by the corrector input for the accelerometer and magnetometer.

**Figure 5:**
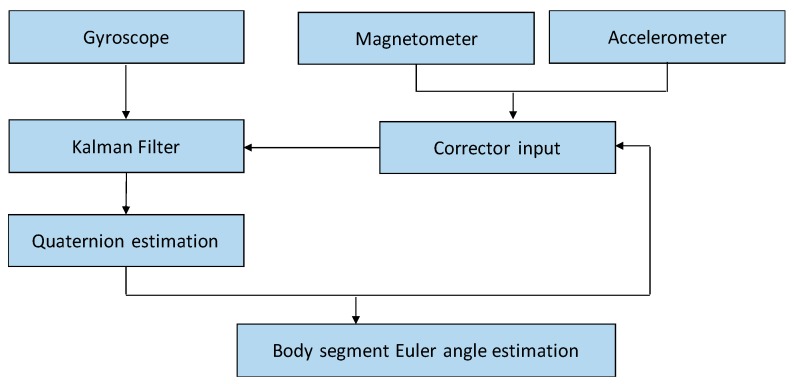
Estimation of the Euler angle of a human segment.

As shown in Figure 5, the Euler angle of a human segment is estimated based on the gyroscope data, accelerometer data, and magnetometer data. The estimation can be achieved based on Yean’s model [28]. A Kalman filter is designed to fuse the sensor data mainly through two stages, namely, applying the filter to the gyroscope data to predict the variables describing the next state and then estimating the orientation by using the accelerometer and magnetometer data to correct the prediction from the gyroscope. In the estimation stage, extracting gravity from the accelerometer doesn’t suffer from the drift issue, meanwhile, the data from the magnetometer measures the calibrated magnetic field values for all three physical axes (*x*, *y*, *z*), especially in the circumstance of the dynamic acceleration and direction movement. These facts enable the correction for the predicted state. The output of the Kalman filter is further used for quaternion estimation to calculate the Euler angle of a human segment and the corrector input by the magnetometer and accelerometer data. Here, quaternion was selected because of its lower computation cost. The quaternion *q* is expressed as (*q_w_*, *q_x_*, *q_y_*, and *q_z_*), where *q_w_* represents its scalar, and *q_x_*, *q_y_*, and *q_z_* represent its vector. Based on the estimated quaternion, calculating the Euler angles of the human segment is easy using the following equation:

(1) $$\left\{ \begin{array}{l} Yaw=atan2(2q_{x}q_{y}-2q_{w}q_{z},2q_{w}^{2}+2q_{x}^{2}-1)\\ Pitch=\arcsin(2q_{w}q_{y}-2q_{x}q_{z})\\ Roll=atan2(2q_{y}q_{z}-2q_{w}q_{x},2q_{w}^{2}+2q_{z}^{2}-1) \end{array}\right.$$

## 4. Fall Detection Algorithm

During a fall activity, the human body first loses balance and falls in a certain direction weightlessly, then impacts against the low objects, such as the ground. In this process, the human posture generally changes from standing to lying or nearly flat. Thus, the process of falls can be divided into three phases as follows: (1) start, the human body lost balance and descents toward the ground; (2) impact, the human body falls to the ground and comes in contact with the ground; and (3) posture, the human body remains lying on the ground for at least a short period of time.

During these three phases, the supporting force for the human body from the ground changes sharply. Thus, the acceleration of the body also changes sharply. Given that the acceleration sensor is triaxial, the acceleration of the human body (RMS) can be calculated from triaxial acceleration components.

**Figure 6:**
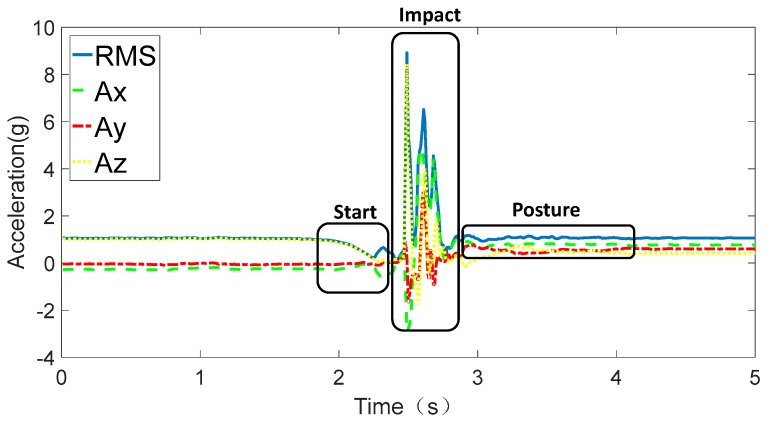
Root mean square (RMS) during a fall activity.

The triaxis accelerometer measures *A_x_*, *A_y_*, and *A_z_* based on forces acting on the accelerometer in the three axes. Thus, RMS will reach 1 g even if the device is stationary due to the influence of gravity. As shown in Figure 6, RMS during the start phase is nearly close to 0 g and changes rapidly to the peak due to the supporting force from the ground during the impact phase. Then, RMS remains almost stable with a slight fluctuation during the posture phase.

**Figure 7:**
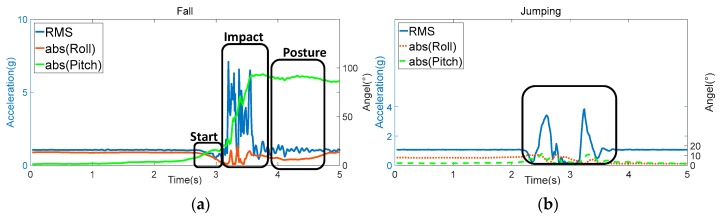
RMS, roll, and pitch during falling and activities of daily living (ADL): (**a**) forward fall ending up lying; (**b**) jumping.

Given that the start phase is not the evident distinction between falls and other activities, such as diving, the fall detection algorithm is based on the recognition of impact and posture phases of the falls, which can be achieved by threshold comparisons of some features. The RMS displays distinctive change in the impact phase of the falls. Thus, RMS can be selected as one of the features for fall detection. When RMS exceeded the acceleration threshold for impact (*A_ti_*), the impact phase is detected. However, only based on the RMS, the fall detection algorithm mistakenly determines some ADLs as falls easily. Some ADL, such as jumping and crouching, may also obtain a similar RMS distribution with falls, as shown in Figure 7. Thus, we detect the posture phase by the Euler angle to achieve precise detection after the impact phase is recognized. Given that the positive and negative values of roll and pitch only represent the direction of the human body, the absolute value of roll and pitch is used to represent the posture of the human body. As shown in Figure 7a, during falls, the posture of the human body changes from standing to lying (or near lying). Therefore, the absolute value of roll (abs (Roll)) or pitch (abs (Pitch)) angles change from nearly 0° to approximately 90°. Meanwhile, during ADLs, the absolute value of roll and pitch angles have a small change, as shown in Figure 7b. Thus, the roll and pitch can be used for posture phase detection through the comparison with orientation threshold of posture (*O_tp_*). If the absolute value of roll or pitch exceeds *O_tp_* within *T_tp_* after the impact phase (the time threshold for posture), the posture phase is detected. After the recognition of the impact and posture phases, a fall of the human body is detected. Given that the recognition of impact and posture phases is based on threshold comparisons, the fall detection algorithm displays good performance in real-time fall detection. Once a fall occurs and the RMS and Euler angle exceed the thresholds, the fall is detected immediately.

Meanwhile, the determination of the threshold for recognizing the impact and posture phases is very important. As proposed by Pierleoni et al. [24], the impact phase is detected when RMS exceeds 2.5 g. However, the sensitivity of fall detection based on the 2.5 g is not good in the reference [24]. Thus, we conducted a set of preliminary experiments with 5 subjects to determine the threshold, which is then used for validating the detection algorithm. The preliminary experiment has the same protocol with the validation experiment. Our observation from the preliminary experimental data found that the peak RMS of the impact phase is more than 2 g. A_ti_ is re-identified by our pre-experiments and set as 2.3 g in our system. The value of 2.3 g is evaluated on the data from the shoulder and waist segments, which has similar distribution during the fall and ADL activities. For the foot segment, we found that A_ti_ during the range of 2.2 g~4.0 g can reach the best detection performance. To facilitate the experiment implementation, we choose 2.3 g as the same A_ti_ for the shoulder, waist, and foot segments. The thresholds for posture phase recognition is set as *O_tp_* = 50 within *T_tp_* = 2 s by referring to [24] because they have been observed to give good performance in our experiments.

## 5. Fall Detection Experiment

We design and conduct a set of experiments by employing subjects to perform specific activities to test the performance of our system and also verify the best human body location to place the sensor unit. In literature, the previous methods have tried the sensor on different body segments individually, such as neck [26], waist [24,29,30], chest and thigh [31], and ankle [30]. Kangas et al. [32] compared the fall detection performance on the head, waist, and wrist and found obvious differences between them. Because the detection performance differs greatly when the sensor is placed on different body segments, our experiments tested the body segments of shoulder, waist, and foot, concerning the facts of easy portability and low disturbance during the fall activities.

The activities designed in the experiment are based on the reality of the elderly fall in daily life, which includes two categories, namely, falls in daily life and ADL designed as interference activities for falls. As shown in Table 1, we divided 16 activities into two categories as follows: 7 falls and 9 ADLs. In daily life, most falls occur during stumbling on things or slipping on a smooth surface, and then the body falls forward or backward. Alternatively, being hit by something from the left and right sides then losing balance causes the body to fall sideways. Thus, the seven simulated falls are grouped into four subcategories, namely, backward fall, forward fall, lateral left fall, and lateral right fall. Furthermore, sometimes the body would not be lying on the ground horizontally, and they are further subcategorized by concerning the different ending-up conditions in backward and forward falls. ADL is designed to verify whether the fall detection system can evoke a fall alarm correctly. In an ideal case, an ADL should not be detected by the system as a fall. However, in some ADLs, the RMS and Euler angle of a certain body segment may have the same features as falls and then were mistaken as falls, decreasing the accuracy of fall detection. Thus, we need to verify the best body location to place the sensor to avoid misjudgment.

A positive or a negative outcome is expected in all the activities in the experiment protocol. If a fall activity evokes the fall alarm, it will be specified as a true positive outcome, and if an ADL evokes the fall alarm, it will be specified as a false positive outcome. Similarly, it will be specified as a true negative outcome or a false negative outcome if no alarm occurs for ADL or a fall activity. An ideal fall detection system should identify all simulated falls as positive outcomes and all ADLs as negative outcomes.

**Figure 8:**
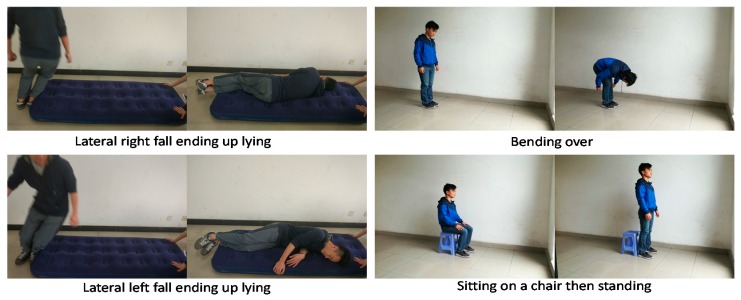
Conducting falling and ADL.

Conducting the experiment would be too risky for elderly users. Thus, a group of 15 healthy subjects aged from 21 to 25 were asked to perform the simulated falls and ADLs (Figure 8). When subjects simulate falls, falling to the ground directly could possibly injure them. Thus, we placed an inflatable mat (thickness 15 cm) on the ground for cushion. Meanwhile, due to the good elasticity of the mat, the subjects would uncontrollably bounce up at a short distance when they fall to the mat. Therefore, the jitter of RMS and Euler angle of the impact are greater than that in the ideal situation.

In our experiment, considering the easy portability, the sensor unit was respectively placed on the subjects’ shoulder, waist, and foot to conduct each activity repeatedly. Scotch tape was used to fix the sensor unit on different segments of the human body. In detail, we fixed the device horizontally on the shoulder (or the foot) by the tape and fixed the device in the coat pocket for the waist segment. The Euler angle displayed a small deviation from the theoretical value because of the irregularities of the human body parts.

Each subject performed all the 16 activities and repeated them after placing the sensor unit on the shoulder, waist, and foot, respectively, and each activity was repeated thrice for each body segment. In the preparation stage, the experimental environment was set up, and the sensor unit was charged to ensure that the battery was adequate during the experiment. Then, the sensor unit was fixed to the participant’s body segment, and the APP was ready to be connected to the sensor unit. In the experiment’s conduction stage, the mat was fixed tightly by two persons to ensure safety, and the participant stood in the front of the mat and then performed all the simulated falls. After the falls, the participant performed all the ADLs. The results during the experiment were recorded by the APP automatically.

We did not collect the data of the same activity sample on the three human segments due to the following concerns: (1) the sensor units simultaneously placed on the three human segments during activities can easily have errors due to the issues of stabilization and inter-disturbance; (2) it is difficult to distinguish which sensor unit issues the alarm when more than one occur, and we need further analysis to verify it. Though there may be difference between the three repeats of an activity on the three human segments, it does not influence the detection results since the different is slight when a subject repeats the same activity. Furthermore, this difference between samples is allowed by our detection algorithm.

## 6. Experimental Results

In the experiment, the RMS, roll, and pitch data of all the participants on the three human body segments (shoulder, waist, and foot) were captured and recorded, respectively, during all the specified simulated falls and ADLs. We found that all the fall activities can be detected correctly when the sensor unit was placed on the three human body segments. However, some ADLs were detected incorrectly when the sensor unit was placed on the shoulder and foot segments.

**Figure 9:**
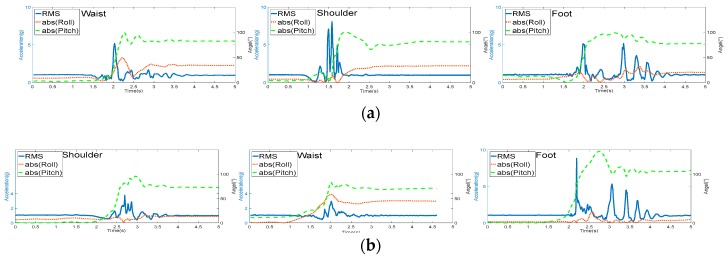

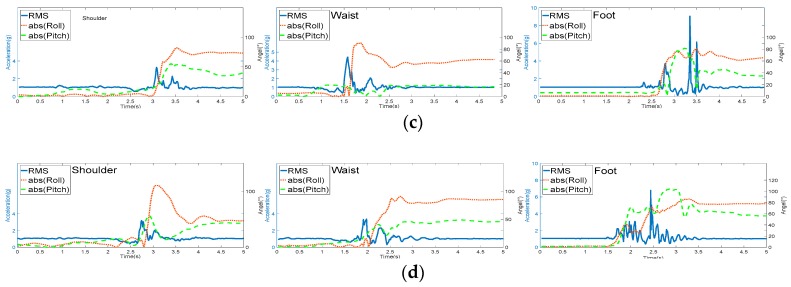
RMS and absolute value of roll and pitch distribution on the shoulder, waist, and foot during typical fall activities: (**a**) Backward fall ending up lying; (**b**) Forward fall ending up lying; (**c**) Lateral right fall ending up lying; (**d**) Lateral left fall ending up lying.

Figure 9 shows the RMS and absolute value of roll and pitch distribution on the three body segments during typical fall activities, including the following: (a) backward fall ending up lying; (b) forward fall ending up lying; (c) lateral right fall ending up lying; and (d) lateral left fall ending up lying. The peaks of RMS distribution on all three body segments exceeds 2.3 g, and the peaks of absolute of roll or pitch exceeded 50° during the simulated falls. Thus, these activities were detected as falls correctly based on the threshold comparison.

**Figure 10:**
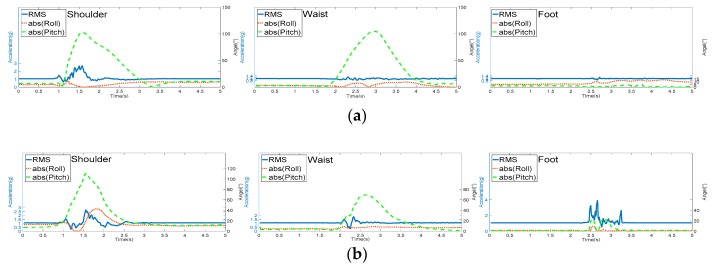

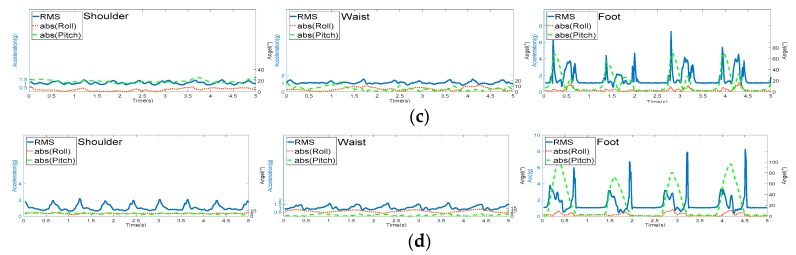
RMS and absolute value of roll and pitch distribution on the shoulder, waist, and foot during ADLs: (**a**) Bending over; (**b**) Staggering forward; (**c**) Walking; (**d**) Taking stairs.

Figure 10 shows RMS and absolute value of roll and pitch distribution on the three body segments during some ADLs: (a) bending over, (b) staggering forward, (c) walking, and (d) taking stairs. Figure 10a,b are detected as falls incorrectly on the shoulder segment because the shoulder sharply changes from near vertical to near horizontal status when the human body is bending over rapidly or staggering forward. In addition, Figure 10c,d are detected as falls incorrectly on the foot segment because the movement of the feet during walking or taking stairs is large compared with that of other human body segments. The peak of RMS and the peak of absolute value of pitch exceed 2.3 g and 50°, respectively, on the shoulder and foot body segments. Meanwhile, in Figure 10a–d, during the same ADLs performed on other segments, the RMS peaks are below 2.3 g and the peaks of roll and pitch are below 50°. The ADLs were detected correctly.

The correct detection rate of these 16 activities including falls and ADL on the three human body segments is summarized in Table 2. This rate is obtained through dividing the number of corrected detections by the total detection samples for an activity implemented by all the subjects. As mentioned in Section 5, the true positive or true negative detection is taken into account as one result of corrected detection. The correct detection rate for all the fall activities is 100%, whereas the detection correct rate in some ADL cases are variable, namely, 60% correct rate for bending over and staggering forward on the shoulder segment. In addition, 6.7% correct rate and 0% correct rate are observed for walking and taking stairs, respectively, on the foot segment.

**Figure 11:**
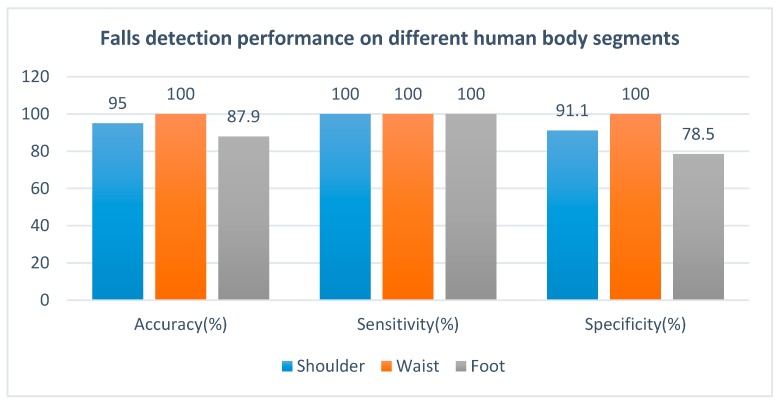
Fall detection performance on different human body segments.

We can further analyze the detection performance of all activities (accuracy), simulated falls (sensitivity), and the ADLs (specificity) to demonstrate the best body location for placing the sensor unit. The accuracy means the total correct detection rate of all the 16 activities on a human segment, in which the sensitivity is the detection correct rate of the 7 simulated falls activities, and the specificity is the detection correct rate of the 9 ADLs. As shown in Figure 11, the sensitivity of detection method is 100% for all three body segments, whereas the specificity of detection method for shoulder, waist, and foot are 91.1%, 100%, and 78.5%, respectively. These values mean that our system has high accuracy of fall detection, and the waist segment is the best location to place the sensor unit, wherein both the detection sensitivity and specificity can reach 100%. During the experiment, all the subjects reach an agreement that the waist is also the most comfortable location to place the sensor unit, which is simply placed in the jacket pocket.

## 7. Discussion and Conclusions

In the literature, there are already many works for human fall detection. To address the advantages of our method, we make a comparison with some previous methods in terms of sensor location, accuracy, portability, and comfort. Though the experiment protocols of these methods are different and the datasets for detection are based on their experiments, all of them considered the distinction between simulated falls and ADLs. In He et al.’s method [26], the acceleration and angular velocity data are measured by a sensor board fixed in the vest and closed to the neck and transmitted to a smart phone. They detect falls based on a Bayes network classifier with an accuracy of 95.67%. In Pierleoni et al.’s method [24], the wearable device is placed on the waist by wearing a belt, and the fall detection is based on the threshold comparison of acceleration and Euler angle, which is similar to our method. However, our method improves the detection accuracy on the waist segment to 100%, benefitting from the threshold of RMS re-identified in our experiment. In Erdogan et al.’s study [29], the data were also measured by wearing a belt on the waist, but a computer is needed to receive and process the data. They used a k-nearest neighbor algorithm to detect falls based on acceleration data at an accuracy of 89.4%. In Ojetola et al.’s method [31], the acceleration and angular velocity data are also measured by wearing a belt on both the chest and right thigh and are then transmitted to a computer. The detection accuracy range is from 98.91% to 99.45%, which is dependent on the training of a machine learning decision tree. In Sorvala et al.’s method [30], the data is measured by wearing a wireless inertial measurement unit (IMU) on the waist and an accelerometer on an ankle. The detection accuracy is 98.69% with 95.6% sensitivity and 99.6% specificity. Yu et al.’s system [33] is vision-based by using a camera and a personal computer, indicating that the fall detection system is limited in the vision field of the camera and the portability is not good. The detection accuracy of their method is 98.13%. Thus, our method has the highest detection accuracy of 100% compared with other methods. Meanwhile, our system has good portability because the small packaged sensor unit is easily fixed in a pocket of a top. For the wearing comfort, all the existing accelerometer sensor-based methods including [24,26] and [29,30,31] depend on the wearing time because long wearing time of a vest or a belt on the waist, thigh, and ankle will lead to discomfort for the user, whereas our method is comfortable to the user, even when porting the sensor in the pocket for a long time.

Our method proposes an easy-to-use, portable human fall monitoring system using only a small self-packaged sensor unit and a mobile phone. We designed 16 simulated falls and ADLs and conducted experiments by placing the sensor units on different body segments, such as the shoulder, waist, and foot of participants. Although fall activities can be correctly detected in all the three body segments, ADL is still possibly mistaken as falls when the sensor unit is placed on the shoulder and foot, but not when placed on the waist. The monitoring system can achieve 100% accuracy, sensitivity, and specificity on the waist segment, verifying that the waist is the best location for placing the sensor. The sensor unit can be placed in the pocket of a top at waist-height without needing to be fixed on the skin. Furthermore, our system is highly portable and comfortable because the sensor unit can be fixed in the pocket of a top and works only with a mobile phone, which offers great portability and will not lead to discomfort during usage. Although we designed the experiment based on the reality of the elderly fall in daily life, the subjects in the experiment are young people to avoid any potential risk of injury. We still need to consider the possible differences in fall activities between the elderly people and young people. Evaluation and validation of the practical application of the proposed system for elderly people is our next objective.

## Figures and Tables

**Table 1:** Falls and ADL designed in the experiment.

Activities		
Fall Activities(FA)	Backward fall	Ending up lying
		Ending up falling to the ground with elbows
		Ending up sitting after lying for 2 s
	Forward fall	Ending up lying
		Falling on the knees, ending up lying
	Lateral left fall	Ending up lying
	Lateral right fall	Ending up lying
Activities of Daily Living (ADL)	Standing-sitting and lying on a mattress-standing	
	Standing-sitting on a chair-standing	
	Taking stairs	
	Bending over	
	Walking	
	Stretching shoulder	
	Staggering forward	
	Staggering backward	
	Twisting waist	

**Table 2:** Detection accuracy of 16 activities on the three human body segments in the experiment.

16 Activities		Shoulder	Waist	Foot
		Correct Rate (%)	Correct Rate (%)	Correct Rate (%)
Falls activities	Backward fall			
	Ending up lying	100	100	100
	Ending up falling to the ground with elbows	100	100	100
	Ending up siting after lying for 2 s	100	100	100
	Forward fall			
	Ending up lying	100	100	100
	Falling on the knees ending up lying	100	100	100
	Lateral left fall			
	Ending up lying	100	100	100
	Lateral right fall	100	100	100
	Ending up lying			
ADL activities	Walking	100	100	6.7
	Bending over	60	100	100
	Taking stairs	100	100	0
	Standing-sitting on a chair-standing	100	100	100
	Standing-sitting and lying on a mattress-standing	100	100	100
	Stretching shoulder	100	100	100
	Staggering forward	60	100	100
	Staggering backward	100	100	100
	Twisting waist	100	100	100

## References

[1] World Health Organization (WHO). http://www.who.int/mediacentre/factsheets/fs344/en/.

[2] Heinrich S., Rapp K., Rissmann U., Becker C., König H.H. (2009). Cost of falls in old age: A systematic review. Osteoporos. Int..

[3] Tran T.H., Le T.L., Hoang V.N., Vu H. (2017). Continuous detection of human fall using multimodal features from Kinect sensors in scalable environment. Comput. Methods Progr. Biomed..

[4] Bian Z.P., Hou J., Chau L.P., Magnenat-Thalmann N. (2015). Fall detection based on body part tracking using a depth camera. IEEE J. Biomed. Health Inform..

[5] Tao S., Kudo M., Nonaka H. (2012). Privacy-preserved behavior analysis and fall detection by an infrared ceiling sensor network. Sensors.

[6] Stone E.E., Skubic M. (2015). Fall detection in homes of older adults using the Microsoft Kinect. IEEE J. Biomed. Health Inform..

[7] Liu S.H., Cheng W.C. (2012). Fall detection with the support vector machine during scripted and continuous unscripted activities. Sensors.

[8] Karantonis D.M., Narayanan M.R., Mathie M., Lovell N.H., Celler B.G. (2006). Implementation of a real-time human movement classifier using a triaxial accelerometer for ambulatory monitoring. IEEE Trans. Inform. Technol. Biomed..

[9] Lai C.F., Chang S.Y., Chao H.C., Huang Y.M. (2011). Detection of cognitive injured body region using multiple triaxial accelerometers for elderly falling. IEEE Sens. J..

[10] Kangas M., Konttila A., Winblad I., Jamsa T. Determination of Simple Thresholds for Accelerometry-Based Parameters for Fall Detection. Proceedings of the EMBS 2007 29th Annual International Conference of the IEEE Engineering in Medicine and Biology Society.

[11] Luque R., Casilari E., Morón M.J., Redondo G. (2014). Comparison and characterization of android-based fall detection systems. Sensors.

[12] Li Q., Stankovic J.A., Hanson M.A., Barth A.T., Lach J., Zhou G. Accurate, Fast Fall Detection Using Gyroscopes and Accelerometer-Derived Posture Information. Proceedings of the BSN 2009 Sixth International Workshop on Wearable and Implantable Body Sensor Networks.

[13] Nari M.I., Suprapto S.S., Kusumah I.H., Adiprawita W. A Simple Design of Wearable Device for Fall Detection with Accelerometer and Gyroscope. Proceedings of the 2016 International Symposium on Electronics and Smart Devices (ISESD).

[14] Andò B., Baglio S., Lombardo C.O., Marletta V. (2016). A multisensor data-fusion approach for ADL and fall classification. IEEE Trans. Instrum. Meas..

[15] Yang L., Ren Y., Hu H., Tian B. (2015). New fast fall detection method based on spatio-temporal context tracking of head by using depth images. Sensors.

[16] Gasparrini S., Cippitelli E., Spinsante S., Gambi E. (2014). A depth-based fall detection system using a Kinect® sensor. Sensors.

[17] Lee Y.S., Chung W.Y. (2012). Visual sensor based abnormal event detection with moving shadow removal in home healthcare applications. Sensors.

[18] Anderson D., Keller J.M., Skubic M., Chen X., He Z. Recognizing Falls from Silhouettes. Proceedings of the 2006 International Conference of the IEEE Engineering in Medicine and Biology Society.

[19] Rougier C., Meunier J., St-Arnaud A., Rousseau J. Monocular 3D Head Tracking to Detect Falls of Elderly People. Proceedings of the 2006 International Conference of the IEEE Engineering in Medicine and Biology Society.

[20] Munoz-Organero M., Lotfi A. (2016). Human movement recognition based on the stochastic characterization of acceleration data. Sensors.

[21] Hsieh C.Y., Liu K.C., Huang C.N., Chu W.C., Chan C.T. (2017). Novel hierarchical fall detection algorithm using a multiphase fall model. Sensors.

[22] Kurniawan A., Hermawan A.R., Purnama I.K. E. A Wearable Device for Fall Detection Elderly People Using Tri Dimensional Accelerometer. Proceedings of the 2016 International Seminar on Intelligent Technology and Its Applications (ISITIA).

[23] Albert M.V., Kording K., Herrmann M., Jayaraman A. (2012). Fall classification by machine learning using mobile phones. PLoS ONE.

[24] Pierleoni P., Belli A., Palma L., Pellegrini M., Pernini L., Valenti S. (2015). A high reliability wearable device for elderly fall detection. IEEE Sens. J..

[25] Wibisono W., Arifin D.N., Pratomo B.A., Ahmad T., Ijtihadie R.M. Falls Detection and Notification System Using Tri-axial Accelerometer and Gyroscope Sensors of a Smartphone. Proceedings of the 2013 Conference on Technologies and Applications of Artificial Intelligence (TAAI).

[26] He J., Bai S., Wang X. (2017). An unobtrusive fall detection and alerting system based on Kalman filter and Bayes network classifier. Sensors.

[27] Ntalampiras S., Roveri M. An Incremental Learning Mechanism for Human Activity Recognition. Proceedings of the 2016 IEEE Symposium Series on Computational Intelligence (SSCI).

[28] Yean S., Lee B.S., Yeo C.K., Vun C.H. Algorithm for 3D Orientation Estimation Based on Kalman Filter and Gradient Descent. Proceedings of the 2016 IEEE 7th Annual Information Technology on Electronics and Mobile Communication Conference (IEMCON).

[29] Erdogan S.Z., Bilgin T.T. (2012). A data mining approach for fall detection by using *k*-nearest neighbour algorithm on wireless sensor network data. IET Commun..

[30] Sorvala A., Alasaarela E., Sorvoja H., Myllyla R. A Two-Threshold Fall Detection Algorithm for Reducing False Alarms. Proceedings of the 6th International Symposium on Medical Information and Communication Technology (ISMICT).

[31] Ojetola O., Gaura E.I., Brusey J. Fall Detection with Wearable Sensors-SAFE (Smart Fall Detection). Proceedings of the 7th International Conference on Intelligent Environments.

[32] Kangas M., Konttila A., Lindgren P., Winblad I., Jämsä T. (2008). Comparison of low-complexity fall detection algorithms for body attached accelerometers. Gait Posture.

[33] Yu M., Rhuma A., Naqvi S.M., Wang L., Chambers J. (2012). A posture recognition-based fall detection system for monitoring an elderly person in a smart home environment. IEEE Trans. Inf. Technol. Biomed..

